# A Novel PGAP3 Gene Mutation-Related Megalocornea Can Be Misdiagnosed as Primary Congenital Glaucoma

**DOI:** 10.7759/cureus.29387

**Published:** 2022-09-21

**Authors:** Abdulmajeed I Alhaidari, Amani S Albakri, Suzan S Alhumaidi

**Affiliations:** 1 Ophthalmology, King Khaled Eye Specialist Hospital, Riyadh, SAU; 2 Division of Pediatric Ophthalmology, King Khaled Eye Specialist Hospital, Riyadh, SAU; 3 Genetic and Metabolic Unit, King Saud Medical City, Riyadh, SAU

**Keywords:** gpi deficiency, hpmrs4, pgap3 mutation, primary congenital glaucoma, megalocornea

## Abstract

Hyperphosphatasia with mental retardation syndrome 4 (HPMRS4) is a rare autosomal recessive disorder caused by glycosylphosphatidylinositol (GPI) deficiency. GPI deficiency results from a mutation in one of six known genes. Mutation in post-GPI attachment to protein phospholipase 3 gene (PGAP3) is linked to HPMRS4. Patients usually present with dysmorphic features, developmental delay, central hypotonia, and seizure. However, in our case, we report a novel homozygous missense mutation of PGAP3 gene in a female child who presented with megalocornea, which is an unusual clinical presentation for HPMRS4. Megalocornea, in her first days of life, led to a misdiagnosis of primary congenital glaucoma. Later, other common clinical features of HPMRS4 became apparent.

## Introduction

Glycosylphosphatidylinositol (GPI) deficiency is a relatively new group of genetic disorders that constitute a part of congenital glycosylation disorders caused by defects in the cellular glycosylation process.

One of the GPI deficiency outcomes is hyperphosphatasia with mental retardation syndrome (HPMRS) [1]. It is a rare autosomal recessive disorder first described in 1970 [2]. Six known genes are responsible for HPMRS when mutated. Mutations result in six different phenotypes, but they overlap significantly in clinical presentations. Most of the genes are responsible for the synthesis and transfer of anchor proteins (those genes are phosphatidylinositol glycan [PIG] including PIGV, PIGW, PIGY, PIGO), the remaining are responsible for remodeling (post-GPI attachment to proteins phospholipase [PGAP] including PGAP2, PGAP3). PGAP3 gene is involved in lipid remodeling of glycosylphosphatidylinositol-anchored proteins (GPI-APs). Mutation of PGAP3 gene is associated with the phenotype hyperphosphatasia with mental retardation syndrome type 4 (HRMRS4) (MIM # 615716) and was first identified and reported in 2014 [3].

Most reported patients with HPMRS4 presented clinically with dysmorphic features (cleft palate, hypertelorism, broad nasal bridge, short nose, long philtrum), central hypotonia, developmental delay, brain atrophy, seizure, dilated lateral ventricles on brain MRI, elevated alkaline phosphatase. In this case, we present a novel homozygous missense mutation of PGAP3 gene in a Saudi girl who presented with megalocornea that led to a misdiagnosis of primary congenital glaucoma.

## Case presentation

This is a case of a Saudi female child of healthy consanguineous parents with unremarkable antenatal history. She has a healthy older brother. She was born at full-term via spontaneous vaginal delivery with a birth weight of 3.2 kg. She was admitted to the neonatal intensive care unit (NICU) for four days due to a respiratory issue. 

At one month of age, she was referred to our center as a case of congenital glaucoma. She had megalocornea (corneal diameter was 12 mm in both eyes) and was on bimatoprost and dorzolamide. At this stage, we started work-up for the patient to either confirm or rule out primary congenital glaucoma and other differentials of megalocornea like Neuhauser syndrome and Frank-Ter Haar syndrome.

On her first visit, her intra-ocular pressure (IOP) was 13 mmHg in the right eye and 16 mmHg in the left eye (measured with a pneumotonometer), and the cup-to-disc (CD) ratio was 0.25 in both eyes. Her central corneal thickness was 646 microns in the right eye and 600 microns in the left eye. No corneal edema in both eyes. Dorzolamide was stopped after this visit. 

At two months old, her IOP was 13 mmHg in both eyes (measured with tono-pen). Since her IOP was normal, the patient’s family was instructed to stop bimatoprost three weeks before the next visit to be examined under anesthesia.

**Figure 1:**
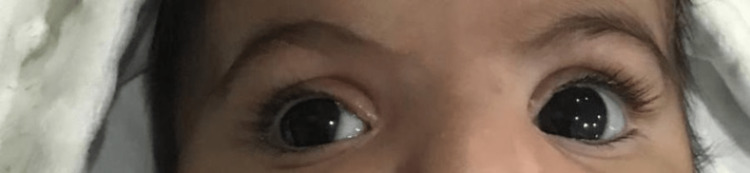
A clinical photo of the patient's eyes at seven months old showing megalocornea.

At seven months old and after stopping bimatoprost temporarily, her IOP was 15 mmHg in both eyes (measured by tono-pen). However, she was not examined under anesthesia. Figure 1 is a clinical photo of the patient's eyes at seven months old showing megalocornea.

At 11 months old, the patient was examined under anesthesia which revealed normal IOP. No corneal edema or Haab striae. No iridodonesis or iris hypoplasia. Axial length (AL) was 21.64 mm in the right eye and 22.18 mm in the left eye. CD ratio of 0.4 in the right eye and 0.3 in the left eye. Normal-appearing angle was appreciated on gonioscopy. Based on this examination, bimatoprost was stopped, and the plan of close follow-up was made.

At one year old, the child was evaluated by a pediatric geneticist and found to be developmentally delayed, not hearing sounds or saying any words, having dysmorphic features with flat occiput, low-set ears, pectus excavatum, central hypotonia, and laryngomalacia. Brain MRI showed mild enlargement of the subarachnoid space and mild dilation of the ventricular system. She had her first seizure attack at that time, and she was later diagnosed with a partial seizure disorder which is controlled with medications.

**Figure 2:**
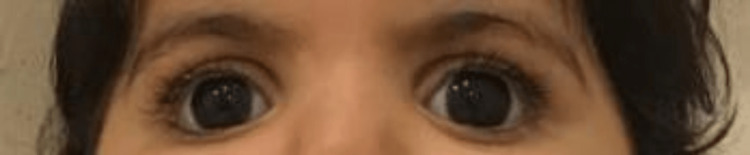
A clinical photo of the patient's eyes at 18 months old showing megalocornea.

At 18 months old, whole-exome sequencing was done and it revealed a novel homozygous missense pathogenic mutation of PGAP3 gene (c.697C>T:p.H233Y). Figure 2 is another clinical photo of the patient's eyes at 18 months old.

At the time of writing this report, the patient is four years old and following up with ophthalmology for her megalocornea and hyperopic astigmatism.

As for her last visit, the mother stated that her daughter was compliant with glasses with better vision. IOP was 17 mmHg in the right eye and 19 mmHg in the left eye. Her refractive status was as follows: for the right eye, the sphere was +2.50, the cylinder was -3.50, and the axis was 180 degrees. For the left eye, the sphere was +2.00, the cylinder was -2.50, and the axis was 180 degrees. A fundus exam showed a flat, large optic nerve head with peripapillary atrophy and blackish pigmentation in both eyes. These myopic changes, despite having hyperopia, are explained by her high AL as it was measured under anesthesia and showed 24.35 mm in the right eye and 24.15 in the left eye. Thus, her hyperopia is mainly cornea-related. 

## Discussion

In this case, we report a novel homozygous missense mutation of the PGAP3 gene associated with an unusual clinical presentation of HPMRS4. Our patient presented at birth with megalocornea which led to a misdiagnosis of primary congenital glaucoma. Despite having megalocornea, primary congenital glaucoma was ruled out due to the normal-appearing angle on gonioscopy, normal IOP, clear cornea with no Haab striae, absence of progressive cupping, visual field defects, buphthalmos, blepharospasm, or photophobia.

Other megalocornea-associated syndromic differentials like Frank-Ter Haar and Neuhauser Syndrome were ruled out because they do not match the clinical picture of our patient.

Frank-Ter Haar disease is mainly a disease of skeletal dysplasia that presents with Brachydactyly and cardiac pathologies, in addition to ophthalmic findings including sunken eyes, hypertelorism, and megalocornea. Neuhauser syndrome is a disease in which patients mainly present with megalocornea, intellectual disability, and motor retardation; in addition to other ophthalmic findings like iridodonesis, iris hypoplasia, nystagmus, and thin corneal central thickness. 

Balobaid et al. delineated the phenotypic spectrum of HPMRS4 in 14 Middle-Eastern patients [4]. They also reviewed the previously published mutations of the PGAP3 gene. We managed to review the novel mutations after their publication date, and the total was 22 different mutations, including our case (Table 1). Three of which were nonsense, 13 were missense, two were small insertion, two were deletion, one mutation was splicing, and one was regulatory.

**Table 1:** Reported PGAP3 gene mutations associated with HPMRS4. PGAP3, post-GPI attachment to proteins phospholipase 3; GPI, glycosylphosphatidylinositol

Mutation	Type of mutation	Ethnicity	Reference
c.203delC (p.C68LfsX88)	Deletion	Lebanese	Abi Farraj et al. [5]
c.265C>T-p.Gln89*	Nonsense	South African	Da’as et al. [6]
c.275G > A, p.G92D	Missense	Pakistani	Howard et al. [3]
c.314C > G, p.P105R	Missense	Saudi Arabian	Howard et al. [3]
c.314C > A, p.P105Q	Missense	Croatian	Sakaguchi et al. [7]
c.320C > T, p.S107 L	Missense	American, British, and Saudi Arabian	Knaus et al. [8]
c.402dup C, p.M135H fs*28	Small insertion	German and Egyptian	Knaus et al. [8]
c.439dupC, p.L147Pfs*16	Small insertion	American	Howard et al. [3]
c.507C>A (p.Tyr169Ter)	Nonsense	Turkish	Doğan et al. [9]
c.511 T > C, p.C171R	Missense	Japanese	Knaus et al. [8]
c.557G>C, p.Arg186Thr	Missense	Qatari	Bezuidenhout et al. [10]
c.558_10G > A	Splicing	German	Knaus et al. [8]
+559 relative to termination codon	Regulatory	French	Knaus et al. [8]
c.697C > T:p.H233Y	Missense	Saudi Arabian	Our case.
c.817_820delGACT, p.D273Sf s*37	Small deletion	Egyptian	Abdel-Hamid et al. [11]
c.842 T > C, p.L281P	Missense	Japanese	Knaus et al. [8]
c.845A > G,D282G p.D282G	Missense	Palestinian	Knaus et al. [8]
c.850C > T,p.H284Y	Missense	Saudi Arabian	Abouelhoda et al. [12]
c.851A > G,p.H284R	Missense	Qatari and Omani	Yavarna et al. [13]
c.861G > T,p.W287C	Missense	French	Knaus et al. [8]
c.914A > G,p.D305G	Missense	British	Howard et al. [3]
c.924C > A,p,Y308X	Nonsense	Middle Eastern	Trujillano et al. [14]

The most commonly reported clinical features of HPMRS are hypertelorism, upslanted palpebral fissures, broad nasal bridge, short nose, seizure disorder, long philtrum, and cleft-palate [3-11,15-16]. There are certain uncommon features that were reported in HPMRS4 such as gum hypertrophy, ear pits, and large anterior fontanelle [9]. Our patient had the same common clinical features, and she had ventricular system dilation in brain MRI, as this finding was reported in previous cases as well. Also, she had megalocornea which was the first clinical sign that led to the misdiagnosis of primary congenital glaucoma. After some time, when her other clinical features became prominent, further investigations revealed the mutation, and she was labeled with HPMRS4. To our knowledge, megalocornea in HPMRS4 was only reported in two previously published reports; both were in Middle-Eastern patients [4, 17]. One of them was published in 2018 and described 14 patients with HPMRS4; five of them had megalocornea [4].

## Conclusions

This is a case of a Saudi female child diagnosed as HPMRS4 with a novel homozygous missense mutation of PGAP3 gene. She presented initially with megalocornea which led to the misdiagnosis of primary congenital glaucoma before other clinical features of HPMRS4 became apparent. Early detection and treatment for primary congenital glaucoma are crucial for better visual outcomes, however, a thorough examination should be done to prevent misdiagnosis that could put a patient at risk of medications' side effects and unnecessary interventions. Also, our case adds to the recent reports that described the incidence of megalocornea among Middle-Eastern patients with HPMRS4.
